# Study of the Solder Characteristics of IGBT Modules Based on Thermal–Mechanical Coupling Simulation

**DOI:** 10.3390/ma16093504

**Published:** 2023-05-02

**Authors:** Jibing Chen, Bowen Liu, Maohui Hu, Shisen Huang, Shanji Yu, Yiping Wu, Junsheng Yang

**Affiliations:** 1 School of Mechanical Engineering, Wuhan Polytechnic University, Wuhan 430000, China; kysdmbylbw@163.com (B.L.); hmhwhqgdx@163.com (M.H.); shisenhuang2023@163.com (S.H.); yushanji202303@163.com (S.Y.); yangjunsheng2008@163.com (J.Y.); 2 School of Materials Science and Engineering, Huazhong University of Science and Technology, Wuhan 430070, China; ypwu@mail.hust.edu.cn

**Keywords:** IGBT solder layer, packaging reliability, interconnection technology, thermal conductivity characteristics, thermal–mechanical coupling

## Abstract

The insulated-gate bipolar transistor (IGBT) represents a crucial component within the domain of power semiconductor devices, which finds ubiquitous employment across a range of critical domains, including new energy vehicles, smart grid systems, rail transit, aerospace, etc. The main characteristics of its operating environment are high voltage, large current, and high power density, which can easily cause issues, such as thermal stress, thermal fatigue, and mechanical stress. Therefore, the reliability of IGBT module packaging has become a critical research topic. This study focuses on the damage of power device solder layers and applies heat transfer theory. Three typical solders for welding IGBTs (92.5Pb5Sn2.5Ag, Sn3.0Ag0.5Cu (SAC305), and nano-silver solder paste) are analyzed using JMatPro software to simulate their characteristics. First, a finite element analysis method is used to simulate the entire IGBT module with ANSYS Workbench platform. The study compares the impact of three types of solders on the overall heat transfer of the IGBT module under normal operation and welding layer damage conditions. The characteristics are analyzed based on changes in the junction temperature, heat flow path, and the law of thermal stress and deformation. The findings indicated that under steady-state working conditions, adjacent chips in a multi-chip IGBT module had significant thermal coupling, with a maximum temperature difference between chip junctions reaching up to 13 °C, and a phenomenon of heat concentration emerged. The three types of solders could change the thermal conductivity and heat transfer direction of the IGBT module to varying degrees, resulting in a temperature change of 3–6 °C. Under conditions of solder layer damage, the junction temperature increased linearly with the severity of the damage. In the 92.5Pb5Sn2.5Ag and Sn3.0Ag0.5Cu (SAC305) solders, the presence of intermetallic compounds (IMCs) led to more stress concentration points in the solder layer, with the maximum stress reaching 7.14661 × 10^7^ MPa and concentrated at the edge of the solder layer. The nano-silver solder layer had the best thermal conductivity, and the maximum thermal deformation under the same conditions was only 1.9092 × 10^−5^ m.

## 1. Introduction

The insulated gate bipolar transistor (IGBT) module is a power semiconductor device that is voltage driven with full voltage control. It is comprised of a bipolar junction transistor (BJT) and a metal-oxide semiconductor field effect diode (MOSFET). This composite device combines the advantages of both the BJT and MOSFET. The BJT provides a reduction in on-state voltage and an increase in current carrying capacity, while the MOSFET has fast switching performance and is easy to drive. Additionally, the IGBT has excellent voltage withstanding capabilities, making it suitable for a wide range of applications [1]. The IGBT module’s versatility allows it to be used in a broad range of applications, not only in consumer products, such as home air conditioning and washing machines, but also in industrial fields, such as smart grid technology, aerospace, and ship drives, etc. IGBT’s working environment is mainly characterized by high voltage, large current, and high power density. Therefore, the reliability of its packaging is crucial for ensuring a stable system operation [2]. Research results have shown that the failure of most IGBT devices is due to local overheating caused by excessive energy, which in turn, leads to catastrophic failure of the device [3,4]. In recent years, with the rapid development of power device technology, the study of IGBT module packaging reliability has become one of the research hotspots in the semiconductor field.

**Figure 1:**
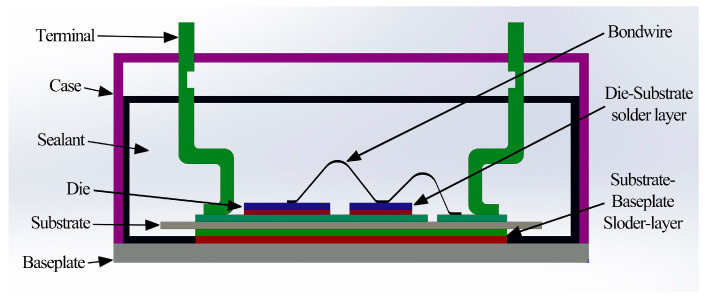
Schematic diagram of IGBT internal structure.

At present, modular IGBT is developed to cater to the needs of high-power environments. IGBT modules have a laminated structure composed of multiple materials, as illustrated in Figure 1. Conventional welding type IGBT module comprises four primary components, including the direct bond copper (DBC) substrate, copper substrate attached to radiator, the IGBT chip, and the fly-wheel diode (FWD). Additionally, aluminum bonding wires, the package housing, and interconnection layers complete the IGBT module [5]. Due to the difference in the coefficient of thermal expansion (CTE) between each layer of material, different magnitudes of thermal stress are generated between the layers, leading to periodic elastic deformation of the bonding surface, which affects the heat dissipation performance and even causes device failure. Despite the high stresses on the chip, DBC board, and substrate, they are well below their respective ultimate tensile stress values. Therefore, this paper focuses on the solder layer, which, in addition to its connecting and supporting function, the stability and performance of the solder layer directly affect the electrical, thermal performance, and long-term reliability of the IGBT module. Therefore, reducing the occurrence of voids and cracks is an important issue in the study of solder layers. Existing literature [6] has established that the solder layer becomes the most vulnerable structure in IGBT module, subject to the complex interplay of electrical, thermal, and heat stress. For instance, Fleischera, A.S. et al. [7] investigated the correlation between the size of the cavity and the change in thermal resistance by the design damage experiment. The authors found that thermal resistance increases linearly with the voidage of random voids and exponentially with the voidage of continuous voids. As a result, fatigue damage of solder layer is the most important reason leading to the failure of power device. According to the available statistics [8,9], the failure accidents of power devices caused by thermal fatigue account for more than 60%. Specifically, when the junction temperature fluctuation range is within 80 °C, the failure of devices is primarily attributable to the damage of the solder layer.

At present, the interconnection technology employed in IGBT modules comprises brazing technology, instantaneous liquid-phase diffusion welding technology, and nano-silver low-temperature sintering technology, as documented in literature [10]. Each welding process exhibits its own set of merits and drawbacks regarding production cost, efficiency, and product quality. Among these, brazing technology is widely employed in electronic packaging as the most prevalent interconnection method. By heating and melting the soft brazing material, the material is capable of wetting and filling the entire gap of the connection layer between the chip and the substrate. Upon cooling and solidifying, a strong bond is formed between the chip and the substrate. The fusible alloy utilized as brazing material facilitates the establishment of a permanent atomic-level connection between the interconnected parts. Hard and soft brazing materials are classified based on their respective melting points. Brazing materials with a melting point over 450 °C are categorized as hard brazing materials, while those with a melting point ranging from 90 to 450 °C are referred to as soft brazing materials, according to literature sources [11]. Considering the high power density and operating temperature of IGBT modules, the selection of brazing materials with high melting points is essential for their packaging. For instance, Sn–Zn brazing materials and Sn–Ag–Cu brazing materials are often utilized due to their high melting points [12].

By the end of the 20th century, the utilization of leaded solders was extensive in major industrial sectors due to their exceptional resistance to oxidation, satisfactory wettability, low melting point, and reasonable cost. The European ROHS and WEEE directives [13] were established to further regulate material and process standards for electronic products from the perspective of human health and environmental protection. Therefore, it also promotes the transition from high-lead solders to lead-free solders, gradually forming a solder system dominated by the tin–silver–copper system. Nano-silver solder paste, apart from its excellent electrical and thermal conductivity and high melting point, presents the benefits of low-temperature sintering and high-temperature service, making it a prime candidate to supplant traditional lead-tin solder in device packaging [14]. A comparative analysis of three low-temperature lead-free brazing alloys, namely, Sn–Zn-based, In-based, and Sn–Bi-based, was presented in literature [15], with a focus on investigating the impact of different elemental compositions on the solder performance. The surface mount process (SMT) was employed as the technical standard in the electronics packaging manufacturing process. Another literature study [16] compared the variations in silver content within the Sn–Ag–Cu solder systems and highlighted the drawbacks of high-silver content solders, such as their high cost, high intermetallic (Ag_3_Sn) content, and poor impact resistance. In contrast, low-silver solders are able to address these limitations by incorporating trace elements to attain a level of overall performance that is comparable to leaded solders, which serves as the key theme and direction of future solder development [17].

Many scholars have studied the IGBT solder layer from different perspectives. Gao W. et al. [18] used finite element analysis to simulate voids in the IGBT solder layer of discrete packaging. Mahmudur M. et al. [19] designed a high-temperature tensile test to study and characterize the mechanical properties of SAC solder doped with different elements at high temperature. The authors of [20] proposed an improved structural thermal model based on the Cauer model to consider the effect of void rate and more accurately describe the thermal conductivity characteristics in the solder layer. This paper mainly explores the properties and thermal conductivity characteristics of the solder layer in the IGBT module packaging, calculates and analyzes the properties of 92.5Pb5Sn2.5Ag and SAC305 solders using JMatPro software (7.0.0, Sente Software, Guildford, United Kingdom), establishes a 3D model of the IGBT and conducts thermal simulation and stress analysis on modules using three different solders: 92.5Pb5Sn2.5Ag, SAC305, and nano-silver, and finally, considers the SAC305 solder as an example to analyze the effect of solder voids on the thermal conductivity and reliability of the IGBT module through simulating the change process of solder voids. The aim is to supplement the research on solder layer material selection, thermal conductivity analysis, and reliability evaluation, in order to provide a strong scientific basis for the improvement of IGBT packaging technology and materials.

## 2. Materials and Methods

The shell of the IGBT module comprises high-temperature resistant PPS material, which possesses numerous merits, including high-heat deflection temperature, minimal molding shrinkage, and excellent dimensional stability. This material provides a sound foundation, stability, and protective effect. Nonetheless, it has poor thermal conductivity. In practice, it is limited by packaging, environment, and operating conditions. In addition, the heat dissipation of thermal radiation and thermal convection are remarkably low, which has little effect on the overall junction temperature. Consequently, inside the IGBT module, heat is primarily conducted from top to bottom in the form of heat conduction, and out through the bottom radiator. The fundamental equation of heat conduction follows Fourier’s law:

(1) $$q=kA\frac{dT}{dx},$$

where k corresponds to thermal conductivity; q represents heat flow density; A indicates the cross-sectional area; and dT/dx denotes the rate of change in temperature.

The differential equations of thermal conductivity in heat transmission are the foundation of the finite element analysis approach used in ANSYS Workbench, utilizing the matrix form associated with the relevant parameters and boundary conditions of the three-dimensional model for numerical calculations.

(2) $$\rho c\frac{\partial t}{\partial \tau }=\varphi \left(\frac{\partial^{2}t}{\partial x^{2}}+\frac{\partial^{2}t}{\partial y^{2}}+\frac{\partial^{2}t}{\partial z^{2}}\right)+\theta ,$$

where ρ represents density; c denotes the specific heat capacity; t indicates the temperature; θ corresponds to the heat generation rate of the IGBT chip; and φ reflects the thermal conductivity.

Supposing that the materials of the layers in the IGBT module within the simulation study are isotropic, the expression for the stress field, derived from the generalized Hooke’s law, is presented as follows:

(3) $$\gamma_{xy}=\frac{\tau_{xy}}{G},\gamma_{yz}=\frac{\tau_{yz}}{G},\gamma_{zx}=\frac{\tau_{zx}}{G},$$

where *G* = e/2(1 + μ); e indicates Young’s modulus; μ represents Poisson’s ratio; $\tau_{xy}$, $\tau_{yz}$, $\tau_{zx}$ denote the shear stress on each of the three surfaces; and $\gamma_{xy}$, $\gamma_{yz}$, $\gamma_{zx}$ symbolize the shear strain on each of the three surfaces.

Due to the discrepancy in thermal expansion coefficients among the material layers, an increase in module temperature results in a rise in thermal stress between the structural layers. Under the influence of alternating temperatures and stresses, the materials become susceptible to creep fatigue and even deformation (e.g., bonding line detachment, solder layer damage, etc.), subsequently increasing the thermal resistance between the material layers and leading to a rise in the module’s overall junction temperature. Consequently, a distinct thermal-force physical field coupling phenomenon is present within the IGBT module. The expressions for the coupling of thermal and force fields in the X, Y, and Z directions are as follows:

(4) $$\begin{array}{l} \varepsilon_{x}=\frac{\partial u}{\partial x}=\frac{1}{e}\left[\sigma_{x}-\mu \left(\sigma_{y}+\sigma_{z}\right)\right]+\alpha \;\Delta T\\ \varepsilon_{y}=\frac{\partial v}{\partial y}=\frac{1}{e}\left[\sigma_{y}-\mu \left(\sigma_{z}+\sigma_{x}\right)\right]+\alpha \;\Delta T\\ \varepsilon_{z}=\frac{\partial w}{\partial z}=\frac{1}{e}\left[\sigma_{z}-\mu \left(\sigma_{x}+\sigma_{y}\right)\right]+\alpha \;\Delta T \end{array},$$

where u, v, w signify displacement; $\sigma_{x}$, $\sigma_{y}$, $\sigma_{z}$ represent thermal stress; $\varepsilon_{x}$, $\varepsilon_{y}$, $\varepsilon_{z}$ denote thermal strain; α corresponds to the coefficient of expansion; and ∆T indicates the variation in temperature between two moments.

**Figure 2:**
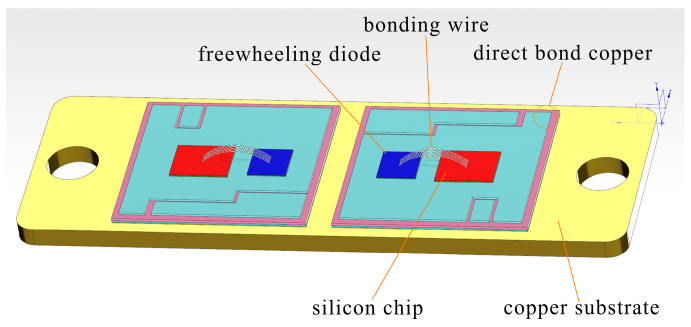
IGBT internal structure model used for simulation experiments.

The IGBT module model FF150R12ME3G serves as the study subject in this paper, and a 1:1 scale IGBT thermal simulation model is created using Solidworks software (2019 sp0.0, Dassault Systemes, Massachusetts, USA), as depicted in Figure 2. The model comprises a structure composed of six layers: The IGBT chip and FWD, the chip solder layer, the base solder layer, the DBC, the ceramic layer, and the copper substrate, and Table 1 presents specific characteristics.

Due to the difference between the simulation process and actual working conditions, in order to highlight the research focus of this paper and considering the computer processing speed and memory capacity, the following settings were made for the simulation process:

- The source of heat is an IGBT chip, and the heat manufacturing is uniform. Thermal radiation’s effect on heat transmission is neglected. The chip’s heat generation rate is loaded, and the chip loss power is set at 100 W and heat generation rate H = P/V = 8 × 10^9^ W/m^3^·K^−1^. The transient thermal simulation was carried out with a heating period of 40 s;
- The emphasis of this study lies on the solder layer within the IGBT, and the effect of the aluminum wire bonding line is disregarded in this simulation due to its minimal impact on the module’s overall junction temperature;
- To simplify the calculation model and more clearly display the heat transfer characteristics of the module, isotropy was assumed for the materials. Since IGBT modules are fixed to the heat sink using bolt tightening in practical applications, fixed constraints were applied to the four corners of the module bottom plate during the mechanical simulation;
- In this simulation procedure, forced convection heat transfer is loaded on the floor and in the surrounding area to imitate the heat dissipation impact of both the thermal grease and the substrate heat sink since they are not modeled. The basal equivalent convective heat transfer coefficient is 3000 W/m^2^·K^−1^, the coefficient of convective heat transport around the substrate is 10 W/m^2^·K^−1^, and the heat flux around the IGBT chip is 1500 W/m^2^;
- During the damage simulation, a circular non-penetration solder void is used to study the influence of void ratio and void radius changes on the junction temperature. To ensure the accuracy of the simulation results, the voids in the solder layer are represented by air properties.

The specific material parameters in the model are shown in Table 2, with the data sourced from the Infineon official website and material handbook.

## 3. Results and Discussion

### 3.1. JMatPro Analysis Results

**Figure 3:**
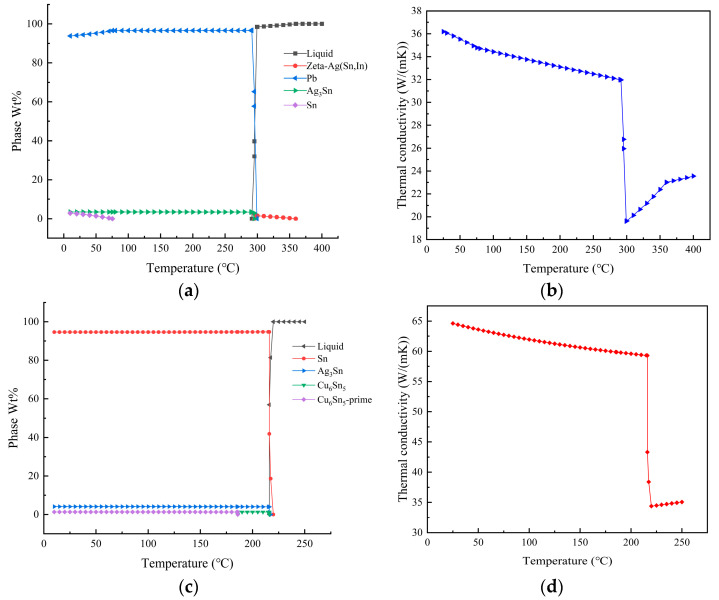
The 92.5Pb5Sn2.5Ag liquid-phase analysis results (**a**) and variation law of thermal conductivity (**b**); SAC305 liquid-phase analysis results (**c**) and variation law of thermal conductivity (**d**).

The temperature-dependent variation of the content of each constituent during the sintering process and the thermal conductivity of the two solders, namely, 92.5Pb5Sn2.5Ag and SAC305, have been illustrated in Figure 3. According to the research in references [21,22] and the calculation results shown in Figure 3a,c, it can be concluded that there are various intermetallic compounds (IMCs) between the lead-based solder and SAC305 solder. Due to the presence of lead, which acts as a solvent material, the formation of metallic bonds between other elements, such as Cu and Sn is facilitated in the liquid state. Consequently, the intermetallic compound (IMC) composition in 92.5Pb5Sn2.5Ag is more complex. However, the higher the IMC content and the more complex the composition, the more susceptible it becomes to failure under the effect of thermal fatigue. The main failure mode is fracture. Despite this, lead is the most stable metal and is less prone to oxidation than other metals. Therefore, the oxidation resistance of the solder joints is greater than that of the SAC305 solder. Moreover, the addition of lead to tin can effectively avoid the transformation of white tin to grey tin, as has been reported in previous studies [23]. During the welding process, the composition of the SAC305 series solder exhibits a more uniform change. Moreover, its liquid-phase line is at 220 °C, which is significantly lower than the liquid-phase line of lead-based solder, which is at 360 °C. This characteristic renders SAC305 solder better wettability and less prone to warping at lower temperatures. Figure 3b,d illustrates that the thermal conductivity of the SAC305 solder is slightly more affected by temperature changes than the lead-containing solder. However, the thermal conductivity of SAC305 solder is two times that of the lead-based solder, making it more suitable for high-power environments. The calculated results are of great significance for the selection and optimization of packaging solders in actual applications of IGBT modules, and can assist in improving the performance and reliability of IGBT modules.

### 3.2. Analysis of the Heat Transfer Characteristics of IGBT Modules

**Figure 4:**
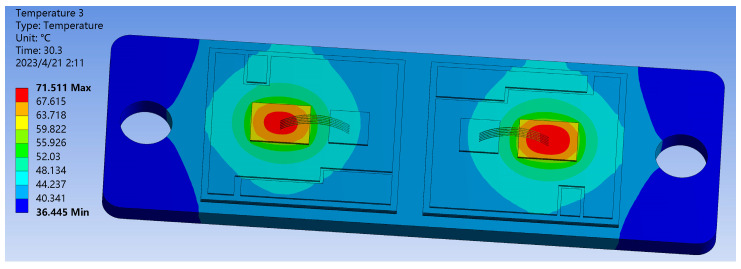
Temperature cloud image of IGBT module during normal operation.

**Figure 5:**
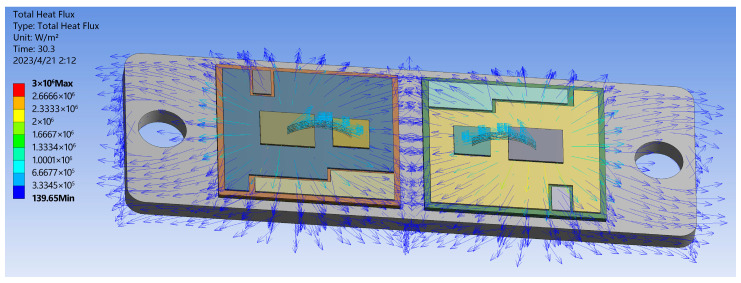
IGBT module heat flow distribution.

**Figure 6:**
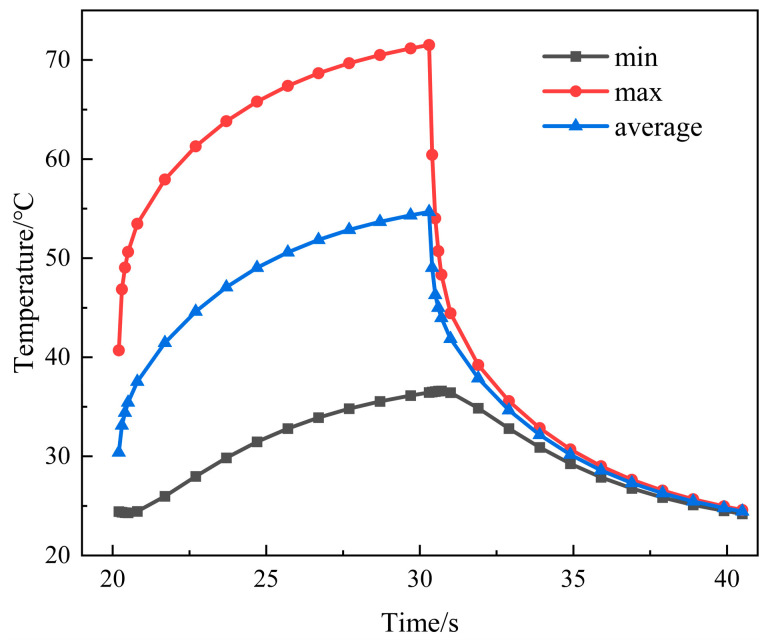
Temperature rise and fall of IGBT module.

In the case of SAC305 solder, the IGBT module’s overall heat transfer is simulated using the transient thermal analysis module. The obtained results are presented in Figure 4 and Figure 5. Combining the simulation results with literature [24], it can be concluded that the heat generated during chip operation mainly transfers from the top to the bottom in an emissive pattern toward the substrate and dissipates through the heat sink. Due to the different thermal conductivity of each layer’s material, the temperature distribution between the internal layers of the module exhibits a gradient variation, which directly affects the distribution of thermal stress within the module. Figure 5 shows the overall heat flow path distribution diagram of the module. The color and density of the arrows in the figure represent the direction and magnitude of the heat flow at that location. The results indicate that the heat transfer between the internal structures of the module is multi-positional, and there is a significant thermal coupling effect between the chips when two or more chips operate simultaneously. As a result of lateral heat transmission, heat tends to concentrate toward the right chip, resulting in the module’s greatest temperature appearing in the middle of the right chip, reaching 71.511 °C. At the copper substrate’s bottom border, the lowest temperature recorded is 36.445 °C. A power cycle simulation is set up using the transient thermal simulation module with a 40 s cycle. The highest and lowest module temperatures during the period of a whole switching cycle are illustrated in Figure 6. The instantaneous conduction and turn-off will cause abrupt changes in the module temperature. Under normal circumstances, the temperature variation follows a stable periodic change with the switching frequency.

### 3.3. Results of the Analysis of the Heat Transfer Characteristics of the Three Solders

**Figure 7:**
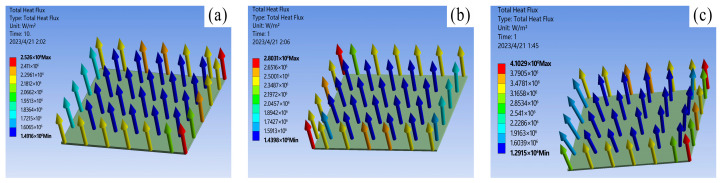
IGBT solder layer heat flux: (**a**) 92.5Pb5Sn2.5Ag; (**b**) SAC305; (**c**) nano-silver solder paste.

**Figure 8:**
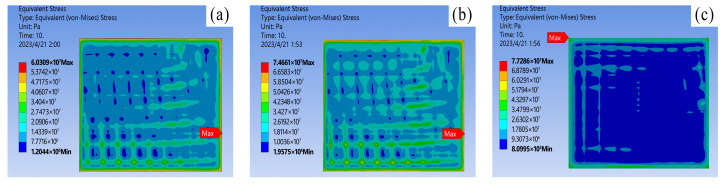
Equivalent stress of solder layer on chip: (**a**) 92.5Pb5Sn2.5Ag; (**b**) SAC305; (**c**) Nano-silver solder paste.

**Figure 9:**
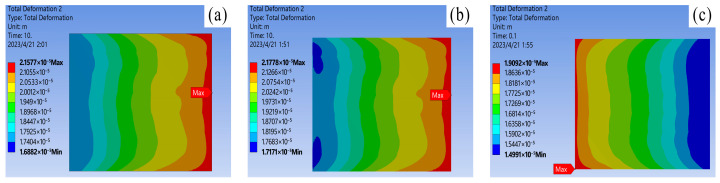
Chip solder layer deformation: (**a**) 92.5Pb5Sn2.5Ag; (**b**) SAC305; (**c**) Nano-silver solder paste.

To guarantee the precision and dependability of the data from the comparative simulation experiments, only the material properties were modified during the simulation process, while other conditions were kept strictly constant. Following steady-state thermal simulation analysis, the junction temperature statistics of the three chip solders of the IGBT module at a power loss of 100 W are presented in Table 3. The results of the thermal-force coupling analysis are depicted in Figure 7, Figure 8 and Figure 9.

Based on the temperature distribution cloud map, it can be concluded that: Under steady-state working conditions, the overall highest junction temperature of the module occurs in the center of the chip. Due to the differences in thermal conductivity between different materials and the effects of thermal resistance stacking at the junctions, the highest junction temperature in the chip solder layer appears at the interface between the solder layer and the chip. The simulation results of the three types of solders show that the thermal conductivity of the nano-silver solder is the best. The overall junction temperature of the module and the junction temperature of the solder layer are both about 4 °C lower under the same operating and cooling conditions. This is attributed to the excellent electrical and thermal conductivity of silver, as well as the uniform composition of the solder material. The heat flux comparison graph for each chip solder layer in Figure 7 shows that the deflection angle of the heat dissipation path with nano-silver solder is smaller than with SAC305 and lead-based solder, resulting in greater heat dissipation efficiency. The data reveal that the heat flux with nano-silver solder paste is 4.1026 × 10^6^ W/m^2^, which is 1.5 times that of the other two solders.

Combining literature [25] and simulation results, it is discovered that under normal operating conditions, the thermal stress does not exceed the yield strength of the material, and the solder layer mainly undergoes elastic deformation. As shown in Figure 8, the variation of equivalent stress in the solder layer: During operation, stress concentration points of varying sizes will appear inside the solder layer, with edge stress being significantly larger than center stress. Among them, the stress concentration phenomenon of nano-silver solder at the edge is the most apparent, with edge stress reaching 7.7286 × 10^7^ Pa. Therefore, the edges and tips of the solder layer are the key areas for strain concentration. Under the action of power cycles, cracks are prone to appear at the edge, and then expand toward the center with fatigue aging. As shown in Figure 9, a comparison of the thermal deformation images of the three solder layers demonstrates that the nano-silver solder has the highest internal stress peak but the lowest deformation, and the distribution is more uniform. The overall degree of deformation is more regular, indicating that the nano-silver solder possesses better resistance to thermal deformation. Moreover, the thermal expansion coefficients of nano-silver, SAC305 solder, lead series solder, and chip materials are 16.51, 22.01, and 26.01, respectively. A comparison of the stress analysis results shows that the greater the difference in CTE, the more irregular the degree of deformation and the change in thermal stress of the material. When designing and manufacturing IGBT modules, it is necessary to select suitable solder materials based on the specific application environment, in order to reduce the junction temperature and improve the reliability of the devices.

### 3.4. Finite Element Investigation of a Cracked IGBT Module Using SAC305 Solder as an Illustration

**Figure 10:**
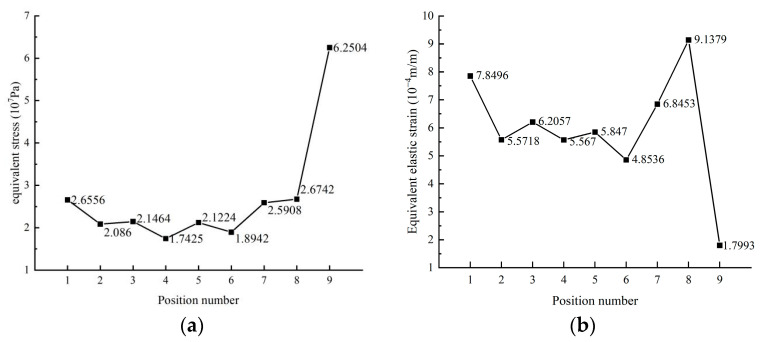
The equivalent stress (**a**) and equivalent elastic strain (**b**) of solder layer change with position.

**Figure 11:**
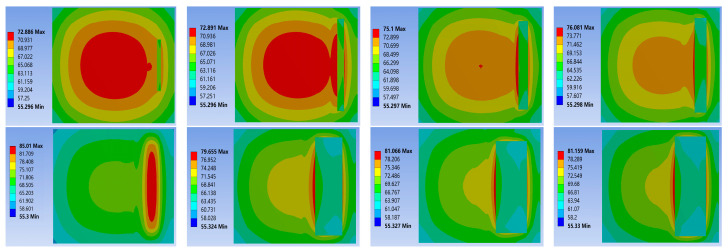
Nephogram of temperature variation in non-penetration hole.

**Figure 12:**
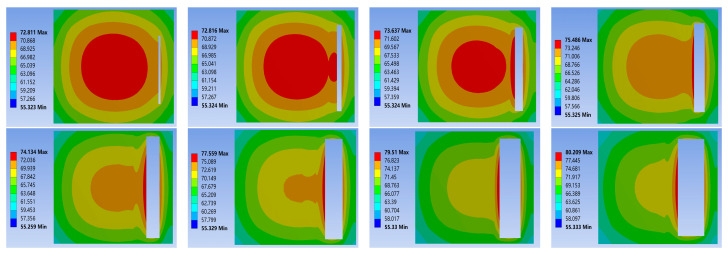
Nephogram of temperature variation in through-hole welding.

There are two main reasons for the formation of voids in the solder layer [26]: 1. During the module’s welding process, air bubbles or incompletely evaporated reflux reactants remaining in the solder can result in cavities within the solder layer’s center; 2. Due to long-term use, fatigue accumulation in the solder layer can lead to the formation of voids of various sizes, which gradually expand. Figure 10 illustrates the way equivalent elastic strain and stress fluctuate from the center of the solder layer through its borders. Owing to the difference in temperature gradients, the curve exhibits a repeated fold, with a general trend of increasing at the edges, where the maximum stress at the edges reaches 6.2504 × 10^7^ Pa, 2.4 times that at the center. From this observation, it can be inferred that voids and cracks tend to initiate at the very edges and occur in the chip’s vertical direction, stretching across the transverse direction, expanding along the longitudinal direction, and progressing toward the center, which is primarily divided into two categories: Penetration and non-penetration. Therefore, in the thermal design of IGBT modules, the heat dissipation effect around the module should be given priority, and the impact of thermal stress should be reduced. This simulation experiment employs SAC305 solder as an example, adopting the form of strip cracks and progressively expanding into a damage model of voids as the void rate increases for research. To guarantee the dependability and correctness of the comparative simulation findings, the 3D model with crack damage was simulated using the same material parameters and power loads as a standard IGBT course simulation. Ten reference values were selected from a cavity rate range of 1–25%, and the fracture depth remained between 8 mm and 12 mm. Figure 11 and Figure 12 present the simulation results.

The existence of cracks or voids will directly alter the heat dissipation path of the module, as indicated by the cloud map of temperature changes in the cracked module displayed in Figure 11 and Figure 12. As the void fraction increases, the voids in the solder layer are replaced by air, greatly reducing the heat dissipation capacity in some areas. There is a significant heat concentration phenomenon at the crack site, and the heat source center continuously shifts toward the void center, resulting in a continuous increase in chip junction temperature and overall junction temperature. A further comparison reveals that the heat source center of non-penetration cracks shifts more quickly, which is due to the air in the through-hole becoming the primary heat conduction medium, while non-penetration voids contain both air and solder as heat conduction media, leading to reduced heat transfer efficiency and more pronounced heat concentration.

**Figure 13:**
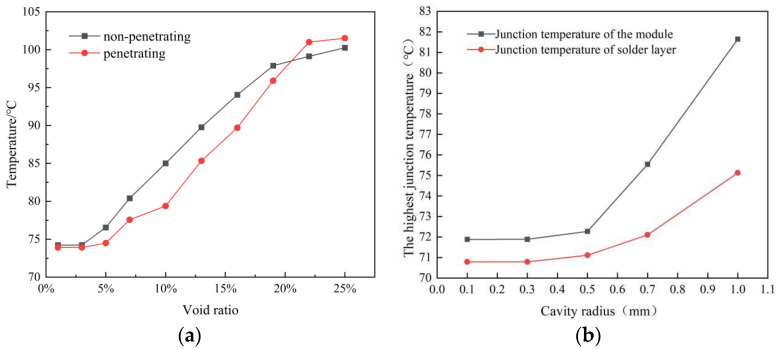
Temperature variation under different cavity forms (**a**); change law of junction temperature and cavity radius (**b**).

Figure 13 shows the results of the junction temperature data obtained from the various experimental groups, which were processed using ORIGIN software (9.8.0.200, OriginLab, Massachusetts, USA). This allows for a clear visualization of the relationship between junction temperature and variations in void ratio and void radius. The results reveal that for void rates of up to 3% or void radii below 0.3 mm, the junction temperature of the solder layer remains largely unchanged from the overall junction temperature. Therefore, it is recommended that the void rate in conventional IGBT modules should be controlled below 2% during factory inspections. As demonstrated in Figure 13a, the average temperature of the solder layer develops linearly with the void rate when the void rate ranges from 5% to 20%. Similarly, Figure 13b shows that as the void radius expands, the rate of junction temperature growth increases, leading to a gradual deterioration in module reliability. In summary, when the void fraction reaches more than 20%, the highest temperature point of the solder layer completely shifts to the void area. At this point, the overall module is in a state of failure or impending failure, which directly affects the performance of the chip.

## 4. Summary

Based on the study of the failure mechanism of IGBT modules, this paper establishes a three-dimensional thermal–mechanical coupled field model. With the help of JMatPro software and ANSYS Workbench simulation platform, computational analysis and finite element simulation methods are used to analyze the thermal conductivity and temperature distribution of three solder materials in IGBT modules, temperature field distribution under steady-state working conditions, the mechanism of thermal stress influence on the chip solder layer, and the effect of crack size and the two types of voids (penetration and non-penetration) on the module’s thermal characteristics. Based on the aforementioned research and in combination with previous studies on the solder layer of IGBT modules, the following conclusions are drawn:

1. The calculation results obtained from JMatPro software show that the 92.5Pb5Sn2.5Ag solder exhibits a higher liquid-phase line compared to the SAC305 solder and possesses a more intricate intermetallic compound (IMC) composition. Conversely, the SAC305 solder demonstrates a higher and more stable thermal conductivity;
2. The normal IGBT module simulation results show that under steady-state working conditions, the internal temperature of the multi-chip IGBT module shows a gradient decrease, with the maximum temperature difference reaching 35.066 °C. Adjacent chips have clear thermal coupling, with a chip junction temperature difference of up to 13 °C. The temperature change is stable with the switching frequency in a periodic manner;
3. The thermal–mechanical coupling simulation results of the solder layer indicate that the nano-silver solder has better thermal conductivity and more uniform internal stress distribution than SAC305 and 92.5Pb5Sn2.5Ag solders. Additionally, it has a lower deformation amount under the same junction temperature conditions and better resistance to thermal deformation under thermal cycling;
4. The damage model simulation results show that the junction temperature and void ratio have a linear relationship within the range of 5% to 20%. The larger the void size, the more apparent the increase in thermal resistance and temperature. It is recommended to maintain the void ratio below 3%. Non-penetration voids have a greater impact on the module’s heat dissipation path than penetration voids, but the size of penetration voids has a more significant effect on the rate of junction temperature change;
5. The research shows that there are differences in the degree of stress concentration in different solders, especially the stress concentration phenomenon around the solder edges. When designing the thermal aspects of IGBT modules, the heat dissipation effect around the module should be considered to reduce the impact of thermal stress. When evaluating the reliability of the solder layer, if cracks are unavoidable due to production conditions or operating environments, efforts should be made to control the location of damage to minimize the impact on the overall reliability of IGBT modules, and thus extending their service life.

## Figures and Tables

**Table 1:** Module size parameters.

Name of Structure	Length/mm	Width/mm	Thickness/mm
Chips	9	7	0.2
Diodes	6	6	0.2
Chip solder layer	9	7	0.12
DBC top/bottom copper layer	30	28	0.3
DBC ceramic layer	30	28	0.38
Substrate solder layer	30	28	0.12
Copper substrates	90	32	3

**Table 2:** Material parameters of the model.

Material Parameters	Density kg/m^3^	Thermal Conductivity W/(mK)	Coefficient of Thermal Expansion 10^−6^ K^−1^	Poisson’s Ratio	Specific Heat Capacity J/(kg·K)	Young’s Modulus MPa
Cu	8960	390	17	0.37	390	110,000
Sn–Ag–Cu	7300	54	25	0.4	230	34,300
AlN	3400	170	4.5	0.22	710	3200
Si	2330	119	2.99	0.28	700	167,000
Air	1.09	2.733			1.017	
Nano-silver	8500	160	19.5	0.25	235	50,000
Pb–Sn–Ag	11,000	35.8	29	0.35	170	24,700

**Table 3:** Junction temperature summary results.

	92.5Pb5Sn2.5Ag	Nano-Silver	SAC305
Global junction temperature	74.562 °C	68.106 °C	71.511 °C
Chip solder lamination temperature	73.461 °C	67.006 °C	70.781 °C

## Data Availability

Not applicable.
